# Undernutrition and its associated factors among school adolescent girls in Abuna Gindeberet district, Central Ethiopia: a cross-sectional study

**DOI:** 10.1186/s40795-022-00587-8

**Published:** 2022-08-24

**Authors:** Segni Mulugeta Tafasa, Meseret Robi Tura, Ermiyas Mulu, Zenebu Begna

**Affiliations:** 1 Department of Public Health, College of Medicine & Health Sciences, Ambo University, P. O. Box 19, Ambo, Ethiopia; 2 Department of Nursing, College of Medicine & Health Sciences, Ambo University, Ambo, Ethiopia

**Keywords:** Adolescent girls, Associated factors, Undernutrition, Abuna Gindeberet

## Abstract

**Background:**

Adolescent is the population whose age between 10–19 years old. They are undergoing rapid growth, development and are one of the nutritionally at-risk groups who should need attention. Adolescent undernutrition is a worldwide problem. Even if this stage brings the second window of opportunity to break the intergenerational cycle of undernutrition little is known specifically in the study area. This study was conducted to assess the prevalence of undernutrition and its associated factors among school adolescent girls in Abuna Gindeberet district, Central Ethiopia, 2021.

**Methods:**

Institution-based cross-sectional quantitative study design was conducted in Abuna Gindeberet district among 10–19 years adolescent girls attending primary and secondary schools from January 1–30, 2021. A systematic random sampling technique was used to select 587 adolescent girls. Data were collected by using interviewer-administered structured and anthropometric measurements. Data were coded, then entered into the Epi-info version 7.2.2.6 and exported to SPSS version 25 and WHO Anthro plus for analysis. Logistic regression analysis was done to identify predictors of under nutrition. Level of statistical significance was declared at *p*-value < 0.05.

**Results:**

The overall magnitude of stunting and thinness were 15.4% [95% CI (12–18)] and 14.2% [95% CI (11–17)] respectively. Number of meals per day [AOR = 3.62, 95% C.I (2.16, 6.05)], adolescent girls of lower grades [AOR = 2.08, 95% C.I (1.07, 4.04)] and who did not begin menstruation [AOR = 1.71, 95% C.I (1.06, 2.73)] were significantly associated with stunting. Adolescent girls engaged in vigorous intensity activities [AOR = 2.51, 95% C.I (1.14, 5.54)], poor dietary diversity score [AOR = 4.05, 95% C.I (1.43, 11.46)] and adolescent age [AOR = 3.77, 95% C.I (1.06, 13.37)] were significantly associated with thinness among adolescent girls.

**Conclusions:**

Adolescent girl's undernutrition is a public health problem in the study area. The number of meals per day, adolescent girls of lower grades and who did not begin menstruation were significantly associated with stunting as well as adolescent girls engaged in vigorous-intensity activities; poor dietary diversity score and adolescent age were significantly associated with thinness among adolescent girls. Therefore, government and other stakeholders should focus on these identified factors to improve the nutritional status of adolescent girls.

## Background

An adolescent is the population whose age between 10–19 years old and it is the period of gradual transition from childhood to adulthood that normally begins with the onset of signs of puberty, which is characterized by important psychological and social changes, not only physiological changes and classified into early adolescence (10–14 years) and late adolescence (15–19 years) [1–3].

Globally, 1.8 billion adolescents account for 16% of the world's population and nearly 90% of them live in low and middle-income countries[4]. According to 2016 Ethiopian Demographic Health Survey (EDHS) in Ethiopia adolescent comprises 24% of the country’s populations [5].

Nutrient requirements increase in adolescence to meet up the demands of pubertal growth. It is the age at which fast growth and development take place secondary to the infancy period. Adolescents gain up to 50% of their adult weight, more than 20% of their adult height, and 50% of their adult skeletal mass during these ages [6]. In addition to this, different factors affect the dietary habits and behaviors of adolescents, including brain development and understanding of matters that might affect health as well as the broader familial, socio-cultural, and economic environment in which adolescent lives, eats, studies, works and plays [7–9].

Malnutrition during adolescence manifests in three broad groups of conditions: undernutrition (wasting, stunting, or chronic undernutrition and thinness or underweight); micronutrient deficiency or excess (inadequate or excessive intake of vitamins or minerals) and overweight or obesity [10]. Undernutrition occurs when people do not eat or absorb enough nutrients to cover their needs for energy and growth, or to sustain a healthy immune system [11].

According to a different report, adolescent undernutrition is a problem in low and middle-income countries (LMIC). The global school-based study conducted in fifty-seven LMIC among 12–15 years adolescents reported an overall prevalence of 10.2% stunting and 5.5% thinness among boys and girls. From this 36.5% and 25.1% of stunting and thinness were reported among girls from Myanmar and Sri Lanka respectively [12]. Similarly, the study conducted in 40 LMIC among adolescent girls reported a prevalence of thinness of 7.63%, with the highest rate in Asia [13]. Some studies in LMIC estimate stunting in girls aged 15–19 years, which ranges from 52% in Guatemala, 44% in Bangladesh to 8% in Kenya, and 6% in Brazil [14]. In Ethiopia, a different study reported that high levels of stunting ranged from 12.2% to 33.2%, and thinness ranges from 8.82% to 32.2% among adolescent girls [15–24].

Poor nutritional status during any stage of an adolescent can have consequences on cognitive development, resulting in decreased learning ability, poor concentration, and impaired school performance and can retard growth and sexual maturations especially in females it is associated with poor reproductive health outcomes [25, 26]. Undernutrition affects the economy of one country directly, as a loss of productivity due to the poor physical condition and indirectly, because of poor cognitive function and learning abilities [27]. Worldwide 16 million girls aged 15–19 years give birth every year [28]. Poor nutritional status of these adolescent girls does have the effect that passes through generations, an undernourished adolescent girl enters pregnancy with poor nutrient storage and gives birth to low birth weight baby, intrauterine growth restricted baby that is more vulnerable to metabolic disorders in adult life [25].

Chronic undernutrition among adolescents is commonly associated with poverty, poor maternal health and nutrition, frequent illness, or improper infant and young child feeding and care in early life [1, 29]. A study done in a different part of the world identified sociodemographic-related factors, dietary practice-related factors, environmental-related factors, and puberty-related characteristics as major associated factors of adolescent undernutrition [15, 16, 20, 21, 30, 31]. Despite this in Ethiopia, most nutrition programs were focusing on early childhood and pregnant mother nutrition by neglecting adolescent girls. Moreover focusing on adolescent's nutrition will help to improve the nutritional status of adolescents themselves, the nutritional situation of future generations, national economies, and break the intergenerational transmission of undernutrition [32]. Even though the nutritional status of the adolescent is paramount important little was known specifically in the study area. Therefore, this study was aimed to provide information on undernutrition and its associated factors among school adolescent girls in Abuna Gindeberet district, Central Ethiopia.

## Methods

### Study setting and period

The study was conducted in Abuna Gindeberet district governmental schools. This is located in the West Shoa zone, Oromia regional state 192 km to the West of Addis Ababa capital city of Ethiopia and 134 km to North of Ambo town zonal capital city. There are eleven 1–4 grades, forty 1–8 grades and seven high schools (9–12 grades), and two 1–4 grades of private schools. The total adolescent girls in Abuna Gindeberet district school were 19,150. The study was conducted from January 1–30, 2021.

### Study design and population

An institution based cross-sectional quantitative study design was conducted. All adolescent girls (10–19 years) attending schools in Abuna Gindeberet district were source population and all randomly selected adolescent girls from randomly selected schools were study populations. All 10–19 years age girls in the schools of Abuna Gindeberet district, and self-reported pregnant and lactating girls were included and excluded from the study respectively.

### Sample size determination and sampling technique

#### Sample size determination

The sample size was calculated by using a single and double population proportion formula for first and second objective respectively. For first objective by considering proportion of undernutrition among adolescent girls from previous study in the Hawzen district schools (thinness = 32.2%) and (stunting = 33.2%) (42), 95% CI, 4% marginal error. From this prevalence of stunting = 33.2% that yield the highest from the two proportions, sample size calculated as:

$$\mathrm{n}=\frac{{({\mathrm{z\alpha }/}_{2})}^{2}\times \mathrm{pq}}{{\mathrm{d}}^{2}}=\mathrm{n}=\frac{{(1.96)}^{2}\times 0.332\times (1-0.332)}{{(0.04)}^{2}}=533$$

where Z α/2 = critical value for normal distribution at 95% confidence level which equals to 1.96; P = prevalence of stunting from study in Hawzen district school, q = 1-p; d = margin of error.

Then by adding 10% non-response rates sample size for first objective became 587.

For the second objective, by using assumption of 80% power, 95% C.I, then by using Epi info statcalc for the most significant variable from previous study.

#### Sample size calculation for second objectives

**Table:** 

S/N	Selected variable	Prevalence among exposed	Prevalence among unexposed	Sample size	With 10% non-response rate
1	Grade level (42)	Prevalence of thinness among grade 4–8 = 16.7% AOR = 2.95	Prevalence of thinness among grade 9–12 = 15.5%	440	484
2	DDS (23)	Prevalence of thinness among low DDS = 15.6% AOR = 2.1	Prevalence thinness among adequate DDS = 5.7%	342	377

From the calculated sample size, sample size for the first objective gave highest which were 587.

### Sampling technique and sampling procedures

First schools were stratified into primary school (4–8 grades) and secondary school (9–12 grades). From forty primary schools, 12 schools (4–8 grades), and seven secondary schools (9–12 grade) three schools were selected by lottery method. The numbers of adolescent girls were obtained from the respective school director's office. From registration girl's age, 10–19 years were screened before actual data collections. The sample size was allocated to each selected school by using probability proportional allocation to total adolescent girls. Then k (constant interval was determined by dividing total number of study population (N) by required sample size(n)). k = N/n = 6101/587 = 10. Then, from 1 to 10 random start were selected by lottery methods. The random start 4 were selected, then every 10^th^ adolescent girls were selected from school registers until fulfilling the required sample size (Fig. 2.)

### Operational and term definitions

Undernutrition is regarded as the presence of stunting and/ or thinness.

Thinness is defined as the proportion of adolescent girls with value BAZ < -2 SDs [33].

Stunting is defined as the proportion of adolescent girls with value HAZ < -2SDs [33].

Dietary Diversity Score is the sum of food groups eaten by adolescent girls over the last 24 h [34].

Meal pattern: the measure of whether they consumed their meal regularly or skipped some times.

Primary school: In this study, institutional schools that have students from grades 4–8.

Secondary school: Institutional schools that have students from grades 9–12.

### Data collection tool and procedure

Data were collected by face-to-face interview using a pre-tested structured questionnaire adapted from previous studies, guidelines prepared by Food and Agricultural Organization, and anthropometric measurements [21, 30, 34, 35]. The adolescent dietary diversity was measured by a qualitative recall of all foods consumed by each adolescent girl during the previous 24 h, which were validated tools prepared by FAO. It is a dichotomous indicator of whether or not to feed ≥ 4 of 9 food groups in the last 24 h. This was categorized as poor dietary diversity score (< 4 food groups) and good dietary diversity score (≥ 4 food groups). The nine food groups considered were starch staples, dark green leafy vegetables, vitamin A-rich fruits and vegetables, other fruits and vegetables, organ meat, flesh meat, eggs, legumes, and nut and milk and milk products (34). Physical activities levels of adolescent girls were assessed by using the WHO STEP wise approach to non-communicable disease risk factor surveillance (STEPS). Which contained four parts related to work-related activities; travel to and from places, recreational activities, and sedentary behavior [36].

#### Anthropometric measurements

After training, data collectors were recorded height and weight by using a portable non-stretchable plastic height-measuring board with a sliding head bar following standard procedures and portable digital scales respectively. For height measurement, subjects were asked to stand erect with their shoulders level, hands at their sides, thighs and heels comfortably together, the buttocks, scapulae, heels, and head were positioned in contact with the vertical backboard with a sliding head bar. Then height was measured to the nearest 0.1 cm.

For weight measurement, adolescent girls were asked to remove their shoes, wear light clothes (schools' uniforms) and then, trained data collectors have weighed the subjects on a calibrated portable digital scale and were record the value to the nearest 0.1 kg. Each measurement was standardized and calibrated by carefully handling, placing the weight scale on a flat surface, and confirmed reading at zero to ascertain accuracy every time before measurements.

### Data quality control

Data were collected by using pretested and structured questionnaires. The one-day training was given to both data collectors and supervisors. Language experts translated questionnaires to Afan Oromo and then back to English to check the consistency. Pretest was performed before actual data collection on 5% of sample size in neighbor district Gindeberet. Height and weight were taken two times to minimize intra and inter observer’s variability of the data collectors, relative technical error measurement (TEM) was calculated. The accepted relative technical measurement error for intra-observer and inter-observer were less than 1.5% and less than 2% respectively. The proper functioning of digital weight scales was checked every time before weight measurement. The data collectors assured the reading scale was exactly at zero before taking weight. Before data entry into the computer, every questionnaire was checked for completeness.

### Data processing and analysis

First, questionnaires were checked for completeness and consistency before data entry. Then, data were coded and entered onto the Epi-info version 7.2.2.6 and exported to SPSS version 25 for analysis. Anthropometric data and other essential variables were exported to WHO Anthro-plus software, a computer program that converts anthropometric data into *Z*-scores of the indices, BAZ and HAZ, by using WHO 2007 population references. Descriptive statistics such as frequency, proportions, mean and standard deviation were used to describe characteristics of the study population. Normality for continuous variables was checked and data were normally distributed. The presence of multicollinearity between independent variables was checked by using the variance inflation factor (VIF). However, there was no identified variable with multicollinearity problems. Bivariable and multivariable logistic regression analysis was carried out to identify predictors of undernutrition among adolescent girls. Model fitness was checked by Hosmer–Lemeshow goodness-of-fit test. An odds ratio with 95% confidence intervals was used to see the strength of association between each independent variable and outcome variable. Level of statistical significance was declared at *p*-value < 0.05*.*

### Ethical consideration

After the briefing, the purposes of the study Research Ethical Committee (REC) of the Ambo University College of Medicine and Health Sciences have ethically approved it. Upon approval letter of permission was obtained from the colleges. For participants, less than 18 years of consent was obtained from their parents and assent from the students. For participants age greater than/equal to 18 years informed consent was obtained from students themselves. Confidentiality and privacy of the information were maintained.

## Results

### Socio-demographic characteristics of study participants

Five hundred eighty-three adolescent girls were included in the study with a response rate of 99.3%. The respondents’ age ranges from 10 to 19 years with the mean age of 14.62 (± 2.38 SD). Around 307 (52.7%) participants were found in the age range of 10 to 14 years. Around 389 (66.7%) and 194 (33.3%) of adolescent girls attend primary and secondary education respectively (Table 1).

**Table 1:** Socio-demographic characteristics of study participants in Abuna Gindeberet district, Central Ethiopia, 2021 (*n* = 583)

Variables		Frequency (*n* = 583)	Percent (%)
Age category	10–14	307	52.7
	15–19	276	47.3
Students Grade	Grade 4–8	389	66.7
	Grade 9–12	194	33.3
Religion	Protestant	431	73.9
	Orthodox	126	21.6
	Others^a^	26	4.5
Place of Residence	Rural	442	75.8
	Urban	141	24.2
Marital status of family	Living together	499	85.6
	Divorced	48	8.2
	Others^b^	36	6.2
Educational status of adolescent father	Have no formal education	226	38.8
	Primary(1–8)	198	34
	Secondary (9–12)	98	16.8
	College and above	61	10.4
Educational status of adolescent mother	Have no formal education	316	54.2
	Primary(1–8)	198	34
	Secondary (9–12)	33	5.7
	College and above	36	6.1
Family size	< 5	83	14.2
	≥ 5	500	85.8

^a^*wakefata, Qaalluu*, ^b^*separated, widowed*

### Environmental related factors

**Fig. 1:**
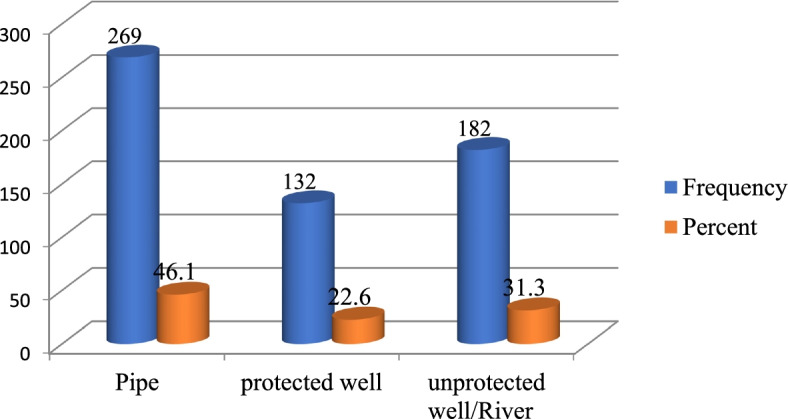
Source of drinking water among school adolescent girls in Abuna Gindeberet district, Central Ethiopia, 2021 (*n* = 583)

Regarding the source of drinking water 269 (46.1%), 132 (22.6%), and 182 (31.3%) were using water from a pipe, protected and unprotected well respectively. 428 (73.4%) of the respondents have a functional toilet in their compound. 222 (38.1%) of study subjects did not wash their hand after visiting the toilet. 235 (40.3%) of adolescent girls had information on adolescent nutrition. The sources of information were from schools 125 (21.4%), HEW 62 (10.7%), and mass media 48 (8.2%) respectively (Fig. 1 and Table 2).

**Table 2:** Environmental related characteristics study participants in Abuna Gindeberet district, Central Ethiopia, 2021 (*n* = 583)

Variables		Frequency (*n* = 583)	Percent (%)
Having home gardening	No	310	53.2
	Yes	273	46.8
Purpose of having home gardening	For sale	7	1.2
	For consumption	121	20.7
	Both	145	24.9
Having functional latrine	No	155	26.6
	Yes	428	73.4
Hand washing after visiting toilet	No	222	38.1
	Yes	361	61.9
Attending mass media	No	199	34.1
	Yes	384	65.9
Having information on adolescent nutrition	No	348	59.7
	Yes	235	40.3
Source of information	Schools	125	21.4
	mass media	48	8.2
	HEW/HP	62	10.7

*HEW* Health Extension Workers, *HP* Health Professionals

### Dietary practice related factors

The mean dietary diversity score of respondents was 4.78 (± 1.22 SD). Among the participants, 503 (86.3%) and 80 (13.7%) had good and poor dietary diversity scores respectively. The most common staple food among study subjects was teff 378 (64.8%), sorghum 93 (16%), maize 79 (13.5%), and wheat 33 (5.7%). Around 292 (50.1%) of the participant had eaten greater than or equal to three meals per day (Table 3 and 4).

**Table 3:** Food groups eaten in the last 24-h among school adolescent girls in Abuna Gindeberet district, Central Ethiopia, 2021 (*n* = 583)

Variables		Frequency (*n* = 583)	Percent (%)
Starchy staple		578	99.1
Dark green leafy vegetables		300	51.5
Vitamin A rich fruits and vegetables		260	44.6
Other fruits and vegetables		355	60.9
Eat organ meat		79	13.6
Flesh meat		115	19.7
Eggs		254	43.6
Legumes/ nuts		488	83.7
Milk and milk products		361	61.9
Dietary diversity score	Poor	80	13.7
	Good	503	86.3

**Table 4:** Food frequency and meal pattern of school adolescent girls in Abuna Gindeberet district, Central Ethiopia, 2021 (*n* = 583)

Variable		Frequency (*n* = 583)	Percent (%)
Eating pattern of diversified meals	Monotonies diet	64	11.0
	Sometimes different	335	57.4
	Always different	184	31.6
Frequency of eating meal per day	≥ 3	292	50.1
	˂ 3	291	49.9
Skipping meals in previous week	No	312	53.5
	Yes	271	46.5
Type of foods	Never	1–2 times per week	≥ 3 times per week
	Frequency (%)	Frequency (%)	Frequency (%)
Frequency of eating cereals	11(1.9)	106(18.2)	466(79.9)
Frequency of eating vegetables	110(18.9)	315(54)	158(27.1)
Frequency of eating tubers and root	41(7)	247(42.4)	295(50.6)
Frequency of eating fruits	303(52)	245(42)	35(6)
Frequency of eating meats	442(75.8)	127(21.8)	14(2.4)
Frequency of eating eggs	303(52)	251(43)	29(5)
Frequency of eating legumes	48(8.2)	149(25.6)	386(66.2)
Frequency of eating milk product	151(25.9)	229(39.3)	203(34.8)
Frequency of eating oil and fats	172(29.5)	272(46.7)	139(23.8)

### Menstrual, illness history, and life style related factors

Out of 583 respondents, 400 (68.6%) of them started to see menstruation. The mean age of menarche was 13.71 (± 0.978SD). About 153 (26.2%) of the participant had a history of illness two weeks before data collection. Regarding the level of physical activities, 222 (38.1%) and 324 (55.6%) of adolescent girls were engaged in vigorous and moderate-intensity activities respectively (Table 5).

**Table 5:** Menstrual, Illness history and Life style related characteristics among school adolescent girls in Abuna Gindeberet district, Central Ethiopia, 2021 (*n* = 583)

Variables		Frequency (*n* = 583)	Percent (%)
Menstrual related factors			
Have you begun menstruation	No	183	31.4
	Yes	400	68.6
Experience premenstrual syndrome	No	208	35.7
	Yes	192	32.9
History of illness in the last two weeks	No	430	73.8
	Yes	153	26.2
Physical activity related characteristics			
Engaged in vigorous-intensity activity	No	361	61.9
	Yes	222	38.1
Engaged in moderate intensity activity	No	259	44.4
	Yes	324	55.6
walk or use a bicycle for at least 30 min continuously to get to and from school	No	307	52.7
	Yes	276	47.3
Engaged in vigorous intensity sports	No	480	82.3
	Yes	103	17.7
Engaged in moderate-intensity sports	No	529	90.7
	Yes	54	9.3

### Magnitude of undernutrition

**Fig. 2:**
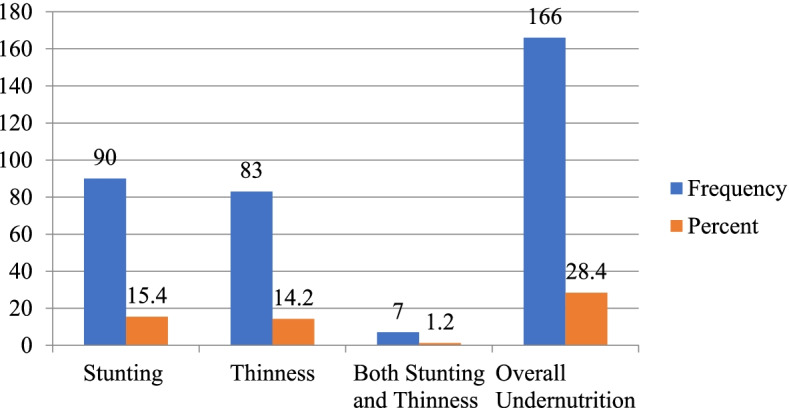
Magnitude of undernutrition among school adolescent girls in Abuna Gindeberet district, Central Ethiopia, 2021 (*n* = 583)

The overall magnitude of stunting 15.4% [95% CI = (12–18)] and thinness 14.2% [95% CI = (11–17)] among school adolescent girls in Abuna Gindeberet district. Around seven (1.2%) of adolescent girls had both stunting and thinness. About 22 (3.8%) and 15 (2.6%) had severe stunting and thinness respectively (Fig. 2).

### Factors associated with stunting among school adolescent girls

Bivariable and multivariable logistic regression analyses were carried out to identify predictors of stunting. On multivariable analysis number of meals per day, level of grade attended and menstruation status were significantly associated with stunting. This study showed that adolescent girls who ate less than three meals per day were 3.62 times more likely to develop stunting when compared to their counterparts [AOR = 3.62, 95% C.I (2.16, 6.05)]. Adolescent girls of lower grades (4–8 grades) were 2 times more likely to develop stunting when compared to adolescent girls of higher grades (9–12 grades) [AOR = 2.08, 95% CI (1.07, 4.04)]. Adolescent girls who did not begin menstruation were 1.71 times more likely to develop stunting when compared to those who started to see menstruation [AOR = 1.71, 95% C.I = (1.06, 2.73)] (Table 6).

**Table 6:** Bivariable and Multivariable result of factor associated with stunting among school adolescent girls in Abuna Gindeberet district, Central Ethiopia, 2021

Variables		Stunting		COR (95% CI)	AOR (95% CI)	*P*-Value
		Yes	No			
Water source	Unimproved	34	148	1.42(0.89, 2.26)	1.79(0.87, 3.68)	0.112
	Improved	56	345	1	1	
Information on adolescent Nutrition	Yes	43	192	1.43(0.91, 2.25)	1.15(0.6, 2.21)	0.674
	No	47	301	1	1	
Patterns of diversified meals	Monotonies	16	48	4.05(1.85,8.88)*	3.12(1, 9.73)	0.05
	Sometimes different	60	275	2.65(1.44,4.89) *	1.34 (0.63,2.84)	0.450
	Always different	14	170	1	1	
Moderate intensity sport	Yes	5	49	0.53(0.21, 1.38)	0.14(0.02, 1.10)	0.062
	No	85	444	1	1	
History of illness	Yes	18	135	0.66(0.38, 1.15)	0.68(0.31, 1.48)	0.329
	No	72	358	1	1	
Begin menstruation	No	39	144	1.85(1.17, 2.94)*	1.71(1.06,2.73)	0.027
	Yes	51	349	1		
Number of meals per day	< 3	68	223	3.74(2.24, 6.25)*	3.62(2.16,6.05)	0.001
	≥ 3	22	270	1	1	
Grade level	4–8 Grades	68	321	1.66(0.99, 2.77)	2.08(1.07,4.04)	0.031
	9–12 grades	22	172	1	1	
DDS	˂4	17	63	1.59(0.88, 2.87)	0.71(0.26, 1.94)	0.506
	≥ 4	73	430	1	1	

^*^Variable with *p*-value < 0.05, *COR* Crude Odds Ratio, *AOR* Adjusted Odds Ratio, *CI* Confidence Interval, *DDS* Dietary Diversity Score

### Factors associated with thinness among school adolescent girls

On multivariable analysis level of activities, poor dietary diversity score and age were significantly associated with thinness among adolescent girls. This study showed that adolescent girls who engaged in vigorous-intensity activities were 2.51 times more likely to develop thinness when compared to their counterparts [AOR = 2.51, 95% CI = (1.14, 5.54)]. Adolescent girls who had poor dietary diversity scores were 4 times more likely to develop thinness when compared to adolescent girls with good dietary diversity scores [AOR = 4.05, 95% CI = (1.43, 11.46)]. Adolescent girls in the early stage (10- 14 years) were 3.77 times more likely to develop thinness when compared to adolescent girls in the late stage (15–19 years) [AOR = 3.77, 95% CI = (1.06, 13.37)] (Table 7).

**Table 7:** Bivariable and Multivariable result of factor associated with thinness among school adolescent girls in Abuna Gindeberet district, Central Ethiopia, 2021

Variables		Thinness		COR(95% CI)	AOR (95% CI)	*P*-Value
		Yes	No			
Water source	Unimproved	32	150	1.46(0.90, 2.37)	0.97(0.42, 2.21)	0.933
	Improved	51	350	1	1	
Home gardening	Yes	32	241	1	1	
	No	51	259	1.48(0.92, 2.39)	0.84(0.37,1.87)	0.664
Washing hand after toilet	No	43	179	1.93(1.21,3.08*	0.69 (0.28,1.73)	0.433
	Yes	40	321	1	1	
Attending mass media	No	44	155	2.51(1.57,4.02*	1.59(0.63,4.02)	0.331
	Yes	39	345	1	1	
Information on adolescent nutrition	Yes	26	209	0.64(0.39, 1.04)	1.24(0.53,2.87)	0.621
	No	57	291	1	1	
Meal pattern	Monotonies	28	36	9.44(4.53,19.7*	1.78 (0.41,7.69)	0.443
	Sometimes different	41	294	1.69(0.9, 3.2)	1.01(0.38,2.69)	0.983
	Always different	14	170	1	1	
Frequency of eating vegetable and Fruits	Never	24	86	2.87(1.41,5.85*	2.07 (0.72,5.97)	0.178
	1–2 times/week	45	270	1.71(0.91, 3.23)	0.78(0.28,2.19)	0.642
	≥ 3times/week	14	144	1	1	
Frequency of eating dairy Product	Never	28	123	2.08(1.12,3.86*	1.56 (0.54,4.52)	0.415
	1–2 times/week	35	194	1.65(0.92, 2.96)	1.79(0.66,4.83)	0.253
	≥ 3times/week	20	183	1	1	
Frequency of eating oil and fat	Never	148	24	1.89 (0.89, 4)	0.97 (0.28,3.33)	0.960
	1-2times/week	224	48	2.49(1.25,4.97)*	1.98(0.66,5.96)	0.227
	≥ 3times/week	128	11	1	1	
Vigorous intensity Activity	Yes	40	182	1.63(1.02,2.59)*	2.51(1.14,5.54)	0.022
	No	43	318	1	1	
PMS	Yes	28	168	2.18(1.13,4.21)*	1.69 (0.74,3.85)	0.213
	No	15	197	1	1	
Meal per day	< 3	53	238	1.95(1.20,3.15)*	1.2(0.50,2.84)	0.684
	≥ 3	30	262	1	1	
Age	10–14 years	59	248	2.5(1.51, 4.14)*	3.77(1.06,13.37)	0.040
	15-19 years	24	252	1	1	
Grade level	4–8 grades	64	325	1.81(1.05,3.13)*	0.43(0.13,1.50)	0.187
	9–12 grades	19	175	1	1	
DDS	˂ 4	32	48	5.91(3.47,10)*	4.05(1.43,11.46)	0.008
	≥ 4	51	452	1	1	

^*^Variable with *p*-value < 0.05, *COR* Crude Odds Ratio, *AOR* Adjusted Odds Ratio, *CI* Confidence Interval, *DDS* Dietary Diversity Score, *PMS* Premenstrual Syndrome

## Discussion

In this study, we assessed the prevalence of undernutrition and its associated factors among school adolescent girls in Abuna Gindeberet district, Central Ethiopia. We found that 15.4% of stunting among adolescent girls. This is nearly similar to the finding from Adama town, Babile district, Lay Guyint district, and Adwa town which were 15.6%, 15%, 16.3%, and 12.2% respectively [16, 17, 24, 30]. The current finding on stunting was lower than the result from west Bengal, Kavre district, and Bangladesh that were 58.36%, 21.08%, and 46.6% respectively [29, 37, 38]. The reason could be due to differences in the study setting, socio-economic, cultural differences, and study time variation. In which study conducted at west Bengal district and Kavre district included adolescents from rural areas. The study conducted in Bangladesh included both rural adolescent boys and girls in whom the current study involved adolescent girls from rural and urban.

Current findings on stunting were also lower than finding from Wukro, Tigray region, Ethiopia (21.2%), Hawzen, Tigray region, Ethiopia (33.2%), Awash town, Afar region, Ethiopia (22.9%), and Amhara region, Ethiopia (31.5%) [15, 18, 19, 23]. The reason for the difference might be due to differences in socio-demographic characteristics, study setting, sample size, and cultural differences in adolescent care. Our finding was greater than a study conducted in Pakistan that reported 8% of stunting among school students [39]. This could be due to the study variation in time zone, socio-economic status, and cultural differences.

The overall magnitude of thinness was 14.2% in the study area. This finding was in line with finding from Kavre district (14.94%), Amhara region (13.6%), Aksum town (12.6%), Asako district (14.8%), and Goba town (11.9%) respectively [15, 20, 21, 38, 40]. Our finding was greater than finding from Awash town (8.82) [18]. The reason may be due to the urban–rural difference of the study subjects. Which study conducted at Awash town included urban adolescent girls only. In this study majority of the participant were from rural residences, in which different studies reported that adolescent girls from rural were more likely to develop thinness [41]. The other could be the majority of study subjects in Awash town were adolescent girls in a late stage who were less likely to develop thinness [15, 22, 40, 42].

The current finding on thinness was lower than the finding from West Bengal (50.89%) and Bangladesh (42.4%) [29, 37]. The reason is a difference in time zone variation, socio-economic difference, and socio-cultural difference. The study from Adwa town (21.4%), Wukro district (21.6%), Hawzen district (32.2%), and Lay Guyint district (29%) were also greater than our finding [19, 23, 24, 30]. The difference is related to a high prevalence of early childhood undernutrition in the Northern part of Ethiopia [43]. The finding of the study was also lower than the study conducted at East Hararge Babile district (21.6%) [16]. This difference is related to the study setting in which the study conducted at Babile district was a community-based study in which school adolescents had more awareness of nutrition information and healthy nutrition practices than those in the general population.

The study showed adolescent girls who ate less than three meals per day were 3.62 times more likely to develop stunting when compared to their counterparts. Studies conducted at Asako district, Adwa town, and Dangila district supported this finding [21, 30, 31]. Nutrient needs to be increased in adolescence to meet the demands of pubertal growth [8]. When adolescent girls ate less than three meals per day they are practicing irregular meal patterns that mean they are skipping regular meals. Irregular meal pattern adds the burden to adolescent girls, thus irregular meal pattern and increased energy requirement due the fast growth spurt leads to an imbalance between energy demand and intake, which finally resulted to adolescent undernutrition.

Adolescent girls of lower grades (4–8 grades) were 2 times more likely to develop stunting when compared to adolescent girls of higher grades (9–12 grades). This is in line with finding from the Tigray region Hawzen district and Jimma zone Southwest Ethiopia [23, 44]. This could be due to adolescent girls who receive higher education are generally more aware than those with basic or no education on how to use available resources for the improvement of their nutritional status and their families. In addition, they can decide not to participate in activities that may put their health at risk. The other could be adolescent girls of higher grade had nutrition information from their related subjects, this leads to the fact that different studies reported that adolescents with nutrition and health information were less likely to develop stunting [15, 45].

Adolescent girls who did not begin menstruation were 1.71 times more likely to develop stunting when compared to those who started to see menstruation. This finding is in line with the study conducted at west Kenya and Adwa town school [30, 46]. This could be because delays in the menstruation status of adolescents were related to the deterioration of their nutrition status [47].

The study stated that adolescent girls who engaged in vigorous-intensity activities were 2.51 times more likely to develop thinness when compared to those who did not engage in vigorous-intensity activities. This is supported by a study conducted at Garhwali, India [48]. This is because adolescent girls who engaged in different vigorous-intensity activities were in demand of high-energy intake due to high metabolism. By itself, the stage needs additional energy due to fast growth spurt and development. If the energy intake and demand due to their level of activities and growth spurt became incomparable their nutritional status becomes deteriorates which leads to adolescent thinness [49].

Adolescent girls who had poor dietary diversity scores were 4 times more likely to develop thinness when compared to adolescent girls with good dietary diversity scores. This finding is supported by the study conducted at Adama town, Asako district, Goba town, Amhara region, Finoteselam town, Aksum town, and Awash town [15, 17, 18, 20, 21, 40, 50]. This is related to the fact that adolescents with low dietary diversity scores will get inadequate energy and other important nutrient required for normal growth and development.

Adolescent girls in the early stage (10–14 years) were 3.77 times more likely to develop thinness when compared to adolescent girls in the late stage (15–19 years). This is in line with finding from Adwa town, Aksum, Eastern Tigray, and the Amhara region [15, 30, 40, 42]. This is because the early adolescent stage is characterized by a fast growth spurt that needs high energy. Thus, if the requirement for achieving their maximum need is not fulfilled, they will be prone to thinness.

## Conclusion

Adolescent girl's undernutrition is a public health problem in the study area. Number of meals per day, adolescent girls of lower grades and adolescents who did not begin menstruations were significantly associated with stunting as well as adolescent girls engaged in vigorous-intensity activities; poor dietary diversity score and adolescent age were significantly associated with thinness among adolescent girls. Therefore, government body, Adolescent girls, families and non-governmental organization should focus on importance of dietary diversity, frequency of meals per day and increased nutritional requirement during adolescent age to improve adolescent girls’ nutritional status.

## Data Availability

The data and all supporting materials used in the preparation of this manuscript are freely available from the corresponding author at reasonable request.

## Abbreviations

BAZ: Body Mass Index for Age Z- Score
BMI: Body Mass Index
CI: Confidence Interval
DDS: Dietary Diversity Score
EDHS: Ethiopian Demographic and Health Survey
FAO: Food and Agriculture Organization
HAZ: Height for Age Z- Score
IDDS: Individual Dietary Diversity Score
IUGR: Intra Uterine Growth Restriction
SD: Standard Deviation
SPSS: Statistical Package for Social Science
UNICEF: United Nations Children’s Fund
USAID: United States Agency International Development
WHO: World Health Organization
